# Advances in Muscle Research in Health and Disease

**DOI:** 10.3390/cells13201694

**Published:** 2024-10-14

**Authors:** Wan Lee

**Affiliations:** 1 Department of Biochemistry, Dongguk University College of Medicine, 123 Dongdae-ro, Gyeongju 38066, Republic of Korea; wanlee@dongguk.ac.kr; Tel.: +82-54-770-2409; Fax: +82-54-770-2447; 2 Section of Molecular and Cellular Medicine, Medical Institute, Dongguk University College of Medicine, 123 Dongdae-ro, Gyeongju 38066, Republic of Korea; 3 Channelopathy Research Center (CRC), Dongguk University College of Medicine, 32 Dongguk-ro, Ilsan Dong-gu, Goyang 10326, Republic of Korea

## 1. Introduction

Muscle tissue plays a vital role in the maintenance of overall health, contributing to essential body functions such as locomotion, respiration, blood circulation, and metabolic regulation. Muscle homeostasis is a dynamic equilibrium that is sustained by a finely balanced interplay between muscle protein synthesis and degradation. This balance is influenced by several factors, including mechanical stress, hormonal signals, nutritional status, and aging. Disruptions in muscle homeostasis can lead to conditions such as sarcopenia, muscular dystrophies, and other muscle-related diseases, which can significantly impact individuals’ quality of life and increase their risk of comorbidities.

In recent years, muscle research has made remarkable progress, greatly advancing our understanding of the complex molecular mechanisms that drive the development, growth, and regeneration of muscle in both health and disease. The research presented in this Special Issue, entitled “Advances in Muscle Research in Health and Disease”, explores these complexities, providing new insights and potential therapeutic strategies. The featured studies cover a wide range of topics, including the molecular pathways involved in muscle differentiation, the effects of external stimuli on muscle function, and innovative approaches to treating muscle diseases. Furthermore, this Special Issue provides a comprehensive overview of the current state of muscle research and highlights the potential for future advancements.

Several key research areas significantly contribute to our understanding of muscle biology, and we highlight these critical fields in this Special Issue. One of the most crucial areas of focus is myogenesis, the process of muscle formation, which is regulated by a network of myogenic regulatory factors (MRFs) and signaling pathways that control the proliferation and differentiation of muscle progenitor cells. Understanding these mechanisms is essential for developing therapies that promote muscle regeneration and repair, particularly in conditions in which muscle tissue is damaged or degenerative. Another important area of research is the role of muscle as an endocrine organ. Muscles secrete a variety of myokines, including cytokines and peptides that exert autocrine, paracrine, and endocrine effects on various tissues and organs. These myokines play a critical role in regulating metabolism, inflammation, and overall muscle function. Research on myokines has opened new avenues for understanding how muscles communicate with other organs and maintain systemic homeostasis.

The potential use of non-invasive treatments in muscle-related diseases is also a rapidly growing field. Techniques such as low-amplitude pulsed electromagnetic fields (PEMFs) and other physical therapies are being investigated for their ability to enhance muscle function and regeneration without the need for surgical intervention. These approaches offer promising alternatives for patients with conditions such as sarcopenia and muscular dystrophies, where traditional treatments may be limited or ineffective. These studies cover a broad spectrum of research, from the molecular intricacies of muscle differentiation and regeneration to the therapeutic potential of non-invasive treatments. Each article provides valuable insights into the mechanisms that regulate muscle function and highlights innovative strategies for managing muscle-related diseases.

By compiling this diverse body of work, this Special Issue aims to deepen our understanding of muscle biology and inspire further research in this critical field. The findings presented here not only enhance our fundamental knowledge of muscle physiology, but also pave the way for novel therapeutic approaches that could significantly improve individuals’ muscle health and overall well-being.

## 2. Highlights of This Special Issue

### 2.1. WAVE2 as a Regulator in Myogenic Differentiation

The first article (contribution 1), authored by Nguyen et al., investigates the pivotal role of WAVE2 in the myogenic differentiation of progenitor cells via the mechanosensitive MRTFA–SRF axis. WAVE2, an actin-binding protein that is essential for actin polymerization, is shown to be critical in skeletal myogenesis. This study demonstrates that the expression of WAVE2 is induced during myogenic differentiation and is crucial for actin cytoskeletal remodeling in C2C12 myoblasts. The knockdown of WAVE2 results in reduced filamentous actin (F-actin) levels, an increased accumulation of globular actin (G-actin), and the impaired nuclear translocation of MRTFA. Consequently, the depletion of WAVE2 inhibits the expression and transcriptional activity of SRF, suppressing myogenic regulatory factors and hindering the differentiation of myoblasts. The mechanisms via which WAVE2 exerts its effects involve the regulation of actin dynamics, which are crucial for the proper functioning of myogenic transcription factors. WAVE2 interacts with the Arp2/3 complex to promote the formation of branched actin networks that are essential for cell migration and morphology. By facilitating actin polymerization, WAVE2 ensures the proper translocation of MRTFA into the nucleus, where it can activate the SRF-dependent transcription of myogenic genes. This study highlights the importance of actin cytoskeletal dynamics in muscle differentiation and provides new insights into the molecular mechanisms underlying this process.

### 2.2. Anticancer Potential of Muscle Secretome Induced by PEMFs

The second article (contribution 2) by Tai et al. explores the anticancer potential of the secretome derived from muscle cells exposed to low-amplitude pulsed electromagnetic fields (PEMFs). The research demonstrates that the brief exposure of C2C12 myotubes to PEMFs generates a conditioned media (pCM) with significant anticancer properties, unlike the conditioned media obtained from untreated cells (cCM). The pCM notably reduces the growth, migration, and invasiveness of breast cancer cells in vitro and diminishes the volume and vascularity of breast cancer microtumors in the chorioallantoic membrane (CAM) model. Furthermore, it was found that blood serum from PEMF-exposed or exercised mice exhibits similar anticancer properties, highlighting the physiological relevance of the findings. The key findings of this study include the identification that High-Temperature Requirement A1 (HTRA1) is crucial for the anticancer efficacy of pCM. HTRA1 is upregulated in response to PEMFs and its presence is necessary and sufficient for the observed anticancer effects. Recombinant HTRA1 mimics the anticancer effects of pCM while its depletion nullifies these effects. Mechanistically, PEMFs promote the release of HTRA1 and other myokines, contributing to muscle myogenesis and anticancer responses. This study suggests that PEMFs induce beneficial muscle adaptations that are similar to those induced by exercise, providing a non-invasive alternative for enhancing muscle secretome responses in therapeutic applications.

### 2.3. Therapeutic Potential of ALY688 in Treating Duchenne Muscular Dystrophy

The third article (contribution 3) by Dubuisson et al. investigates the therapeutic potential application of ALY688, a small peptide adiponectin receptor (AdipoR) agonist, in treating Duchenne muscular dystrophy (DMD). This study addresses the limitations of direct adiponectin (ApN) use due to its complex structure and rapid turnover. ALY688, derived from the active site of human globular ApN, selectively activates AdipoR1, which is primarily expressed in skeletal muscle. The research, which was conducted on four-week-old mdx mice treated with ALY688 for two months, a model for DMD, demonstrated significant improvements in muscle function. The treated mice showed increased force and endurance, and reduced muscle inflammation, oxidative stress, and fibrosis. The key findings include a decrease in pro-inflammatory cytokines (IL-1β, TNFα), oxidative stress markers (HNE, PRDX3), and macrophage infiltration (CD68). ALY688 also mitigated myonecrosis by inhibiting necroptosis, as indicated by the reduction in P-RIP protein levels. Enhanced muscle regeneration was observed through increased dystrophin-positive revertant fibers (RFs) and elevated markers of muscle differentiation (Mrf4, Myogenin, Myh7). Furthermore, ALY688 reduced the levels of fibrosis-related factors (TGF-β, P-Smad2, COL1A1, COL3A1) and activated the AMPK-PGC-1α axis, which suppressed the activity of NF-κB and upregulated Utrophin. This study underscores ALY688’s potential use as a therapeutic in the management of DMD, potentially enhancing muscle function, reducing inflammation, oxidative stress, and fibrosis, and promoting muscle regeneration. These findings offer valuable insights into the molecular mechanisms underlying these beneficial effects, providing a foundation for future therapeutic applications.

### 2.4. Transcriptomic Landscape of Muscular Sarcoidosis

The fourth article (contribution 4) by Lequain et al. employs spatial transcriptomics to explore the transcriptomic landscape of muscular sarcoidosis, a condition characterized by the infiltration of non-caseating granulomas into skeletal muscle. This study analyzed muscle biopsies from two patients, revealing five distinct transcriptomic clusters: perigranuloma tissue (PGT), granuloma structures (GS), and three muscle areas (proximal, intermediate, and distal) relative to the granuloma. The study’s key findings include the identification of granulomas predominantly composed of monocytes/macrophages and T-lymphocytes with significant cytokine activity. Granulomas generate gradients of inflammation and fibrosis, impacting the surrounding muscle tissues. The upregulated pathways include TNF-α, TGF-β, and interferons, promoting fibrosis and inhibiting the regeneration of muscle. Granulomas exhibit a profibrotic macrophage profile similar to that observed in Duchenne muscular dystrophy, marked by the expression of fibrogenesis-related genes such as LGALS3, SPP1, and TREM2. This research highlights the potential of granulomas to induce fibrosis and disrupt muscle homeostasis, suggesting that early and aggressive treatment strategies should be implemented to prevent fibrosis and maintain muscle function. The spatial transcriptomics approach provides valuable insights into the cellular interactions and pathophysiological mechanisms implicated in muscular sarcoidosis, offering a foundation for future therapeutic interventions.

### 2.5. Lysine’s Role in Myogenic Differentiation in Fast and Slow Muscles

The fifth article (contribution 5) by Wang et al. explores how lysine (Lys) affects myogenic differentiation in fast and slow muscles and their satellite cells (SCs) in postnatal piglets. Thirty piglets were divided into three diet groups: control (1.31% Lys), Lys-deficient (0.83% Lys), and Lys rescue (0.83% Lys initially then 1.31% Lys). The researchers evaluated the development of muscle and the SC differentiation mechanisms in response to Lys levels. The key findings of this study were that Lys deficiency significantly hinders the growth of both fast (semimembranosus) and slow (semitendinosus) muscles, and that replenishing Lys reverses these effects, underscoring its critical role in muscle development. Lys deficiency increased the expression of myogenin and MRF4 in fast muscles but decreased them in slow muscles. This differential regulation was also observed in the Wnt/Ca2+ pathway, which was activated in fast muscle SCs but suppressed in slow muscle SCs under Lys deficiency. When sufficient Lys was attained, the Wnt/Ca2+ pathway in fast muscle SCs was repressed while it was stimulated in slow muscle SCs, promoting myogenic differentiation. This study reveals that Lys manipulation affects myogenic differentiation and the Wnt/Ca2+ pathway in a muscle-type-dependent manner, highlighting its essential role in muscle growth and development. These findings provide valuable insights into the differential impact of Lys on fast and slow muscles, providing insights regarding the potential application of targeted nutritional interventions in muscle health and disease.

## 3. Perspectives

The articles published in this Special Issue collectively advance our understanding of muscle biology in several critical areas. The role of WAVE2 in actin dynamics and myogenic differentiation underscores the importance of cytoskeletal regulation in muscle development. The discovery of the anticancer effects of the secretome obtained from PEMF-stimulated muscle cells provides new opportunities for non-invasive cancer therapies. The therapeutic potential of ALY688 in treating DMD highlights the promise of targeting adiponectin receptors to mitigate muscle fibrosis and enhance regeneration. The study of muscular sarcoidosis using spatial transcriptomics provides a novel perspective on the pathophysiological mechanisms of this condition, emphasizing the need for early intervention. Finally, the research on lysine’s differential impact on myogenic differentiation in fast and slow muscles offers valuable insights regarding the application of nutritional strategies that could optimize the growth and repair of muscle.

## 4. Conclusions

This Special Issue, entitled “Advances in Muscle Research in Health and Disease”, presents a collection of focused studies that elucidate the complex mechanisms underlying muscle physiology and pathology. The research featured in this Special Issue not only deepens our fundamental understanding of muscle biology, but also paves the way for novel therapeutic strategies that target muscle-related diseases. As we continue to explore the molecular intricacies of muscle function and dysfunction, these studies lay a strong foundation for future research and clinical applications, ultimately improving muscle health and enhancing disease management.

